# Effectiveness and Safety of Direct-acting Antivirals for Treatment of Adolescents With HCV/HIV Coinfection: Real-world Data From Europe

**DOI:** 10.1097/INF.0000000000004271

**Published:** 2024-02-02

**Authors:** Farihah Malik, Siobhan Crichton, Yulia Plotnikova, Inga Latysheva, Anna Samarina, Maria Pokorska-Śpiewak, Marisa Navarro Gomez, Heather Bailey, Claire Thorne, Ali Judd, Anna Turkova, Intira Jeannie Collins

**Affiliations:** * From the UCL Great Ormond Street Institute of Child Health, University College London, London, United Kingdom; † Medical Research Council Clinical Trials Unit, Institute of Clinical Trials and Methodology, University College London, London, United Kingdom; ‡ Irkutsk AIDS Centre, Irkutsk Regional Centre for the Prevention and Control of AIDS and Infectious Diseases (IOC AIDS), Russia; § Republican Clinical Hospital of Infectious Diseases, Saint Petersburg, Russia; ¶ The City HIV Centre, Saint Petersburg, Russia; ∥ Department of Children’s Infectious Diseases, Medical University of Warsaw; Hospital of Infectious Diseases in Warsaw, Poland; ** Pediatric Infectious Diseases, Hospital Gregorio Marañón, IISGM, UCM, CIBERINFEC ISCIII, Madrid, Spain; †† Institute for Global Health, University College London, London, United Kingdom.; Medical University of Warsaw, Poland, Department of Children’s Infectious Diseases; Hospital of Infectious Diseases in Warsaw, Poland; St Petersburg Republican Hospital; St Petersburg Republican Hospital; St Petersburg Republican Hospital; Irkutsk AIDS Centre; Hospital Universitario La Paz, Madrid; Hospital Universitario La Paz, Madrid; Hospital Universitario La Paz, Madrid; Hospital Universitario La Paz, Madrid; Hospital Universitario La Paz, Madrid; Hospital Universitario Doce de Octubre, Madrid; Hospital Universitario Doce de Octubre, Madrid; Hospital Universitario Doce de Octubre, Madrid; Hospital Universitario Doce de Octubre, Madrid; Hospital Universitario Doce de Octubre, Madrid; Hospital Universitario Doce de Octubre, Madrid; Hospital Clínico San Carlos, Madrid; Hospital Clínico San Carlos, Madrid; Hospital Clínico San Carlos, Madrid; Hospital Universitario de Getafe, Madrid; Hospital Universitario de Getafe, Madrid; Hospital Universitario Gregorio Marañón, Madrid; Hospital Universitario Gregorio Marañón, Madrid; Hospital Universitario Gregorio Marañón, Madrid; Hospital Universitario Gregorio Marañón, Madrid; Hospital Universitario Gregorio Marañón, Madrid; Hospital Universitario Gregorio Marañón, Madrid; Hospital Universitario de Móstoles, Madrid; Hospital Universitario Príncipe de Asturias de Alcalá de Henares, Madrid; Hospital Infantil Universitario Niño Jesús, Madrid; Hospital Universitario de Torrejón, Madrid; Hospital Fundación Jiménez Díaz, Madrid; Hospital Universitario Infanta Leonor, Madrid; Hospital Universitario Donostia, Guipúzcoa; Hospital Universitario Cruces, Vizcaya; Hospital Universitario Basurto, Vizcaya; Hospital Insular Materno Infantil, Gran Canaria; Hospital Insular Materno Infantil, Gran Canaria; Hospital Universitario Virgen de la Candelaria, Tenerife; Hospital General, Lanzarote; Hospital General, Lanzarote; Hospital de Poniente de El Ejido, Almería; Hospital Universitario Virgen del Rocío, Sevilla; Hospital Universitario Virgen del Rocío, Sevilla; Hospital Universitario Virgen de la Macarena, Sevilla; Hospital Universitario Virgen de las Nieves, Granada; Hospital Universitario Virgen de las Nieves, Granada; Hospital Regional Universitario, Málaga; Hospital Regional Universitario, Málaga; Complejo Hospitalario Torrecárdenas, Almería; Complejo Hospitalario Torrecárdenas, Almería; Hospital Universitario Puerta del Mar, Cádiz; Hospital Universitario Puerta del Mar, Cádiz; Hospital Universitario Reina Sofía de Córdoba; Complejo Hospitalario Universitario Infanta Cristina, Badajoz; Complejo Hospitalario, Cáceres; Alejandra Méndez (Hospital de Cabueñes, Asturias; Hospital Universitario Central de Asturias; Complejo Hospitalario Universitario, Albacete; Hospital Virgen de la Salud, Toledo; Hospital Virgen del Camino, Navarra; Hospital Universitario Miguel Servet, Zaragoza; Hospital Clínico Universitario Lozano Blesa, Zaragoza; Complejo Hospitalario Universitario, Pontevedra; Complejo Hospitalario Universitario, La Coruña; Hospital Universitario Lucus Augusti, Lugo; Hospital Universitario La Fe, Valencia; Hospital Universitario La Fe, Valencia; Hospital Universitario de San Juan de Alicante; Hospital General Universitario, Valencia; Hospital General, Castellón; Hospital Universitario Marqués de Valdecilla, Cantabria; Complejo Hospitalario, León; Complejo Hospitalario, Zamora; Complejo Hospitalario, Zamora; Hospital Universitario Virgen de la Arrixaca, Murcia; Hospital Universitario Virgen de la Arrixaca, Murcia; Hospital Universitario Virgen de la Arrixaca, Murcia; Hospital Universitario Gregorio Marañón, Madrid; Hospital Universitario La Paz, Madrid; Hospital Universitario La Paz, Madrid; Hospital Universitario La Paz, Madrid; Hospital Universitario La Paz, Madrid; Hospital Universitario Doce de Octubre, Madrid; Hospital Universitario Doce de Octubre, Madrid; Hospital Universitario Doce de Octubre, Madrid; Hospital Universitario de Getafe, Madrid; Hospital Universitario Gregorio Marañón, Madrid; Hospital Universitario Gregorio Marañón, Madrid; Hospital Universitario Gregorio Marañón, Madrid; Hospital Universitario Gregorio Marañón, Madrid; Hospital Universitario Gregorio Marañón, Madrid; Hospital Universitario Gregorio Marañón, Madrid; Hospital Universitario Gregorio Marañón, Madrid; Hospital Universitario Gregorio Marañón, Madrid; Hospital Universitario Gregorio Marañón, Madrid; Hospital Universitario Gregorio Marañón, Madrid; Hospital Universitario Príncipe de Asturias de Alcalá de Henares, Madrid; Hospital Universitario de Torrejon, Madrid; Hospital Universitario Severo Ochoa de Leganés, Madrid; Hospital Universitario Severo Ochoa de Leganés, Madrid; Hospital Universitario Ramon y Cajal, Madrid; Hospital Universitario Ramon y Cajal, Madrid; Hospital Universitario Ramon y Cajal, Madrid; Hospital Universitario Infanta Leonor, Madrid; Hospital Universitario Infanta Leonor, Madrid; Hospital Universitario La Princesa, Madrid; Hospital Universitario Fundacion Alcorcon, Madrid; Hospital Universitario Fundacion Alcorcon, Madrid; Hospital Fundacion Jimenez Diaz, Madrid; Hospital Universitario Puerta de Hierro de Majadahonda, Madrid; Hospital Universitario Puerta de Hierro de Majadahonda, Madrid; Hospital Universitario Donostia, Guipuzcoa; Hospital Universitario Donostia, Guipuzcoa; Hospital Universitario Donostia, Guipuzcoa; Hospital Universitario Cruces, Vizcaya; Hospital Universitario Basurto, Vizcaya; Hospital Universitario Basurto, Vizcaya; Hospital Universitario Central de Asturias; Hospital Universitario Insular, Gran Canaria; Hospital Universitario de Canarias, Tenerife; Hospital Universitario de Canarias, Tenerife; Hospital Universitario de Canarias, Tenerife; Hospital Universitario de Canarias, Tenerife; Hospital Universitario de Canarias, Tenerife; Hospital Universitario de Canarias, Tenerife; Hospital Universitario Doctor Negrin, Gran Canaria; Hospital Regional Universitario, Málaga; Hospital Regional Universitario, Málaga; Hospital Regional Universitario, Málaga; Hospital Regional Universitario, Málaga; Hospital Universitario Virgen del Rocio, Sevilla; Hospital Universitario Virgen del Rocio, Sevilla; Hospital Universitario Virgen del Rocio, Sevilla; Hospital Universitario Virgen de la Macarena, Sevilla; Hospital Universitario Virgen de la Macarena, Sevilla; Hospital Universitario Virgen de la Macarena, Sevilla; Hospital Universitario Virgen de las Nieves, Granada; Hospital Universitario Virgen de las Nieves, Granada; Hospital Universitario Clinico San Cecilio, Granada; Hospital Universitario Clinico San Cecilio, Granada; Hospital Universitario Reina Sofia, Cordoba; Hospital Universitario Reina Sofia, Cordoba; Hospital Universitario Juan Ramon Jimenez, Huelva; Hospital Universitario Juan Ramon Jimenez, Huelva; Hospital Universitario Juan Ramon Jimenez, Huelva; Complejo Hospitalario Universitario, Albacete; Complejo Hospitalario Universitario, Albacete; Complejo Hospitalario Universitario, Albacete; Hospital Universitario, Guadalajara; Hospital Universitario Miguel Servet, Zaragoza; Hospital Universitario Miguel Servet, Zaragoza; Hospital Alvaro Cunqueiro, Pontevedra; Hospital Alvaro Cunqueiro, Pontevedra; Hospital Alvaro Cunqueiro, Pontevedra; Complejo Hospitalario Universitario, La Coruna; Hospital Universitario La Fe, Valencia; Hospital Universitario La Fe, Valencia; Hospital Clinico Universitario, Valencia; Hospital Clinico Universitario, Valencia; Hospital General Universitario, Alicante; Hospital General Universitario, Alicante; Hospital General Universitario de Elche, Alicante; Hospital General Universitario de Elche, Alicante; Hospital General Universitario de Elche, Alicante; Hospital General Universitario de Elche, Alicante; Hospital Clinico, Valladolid; Hospital Clinico, Valladolid; Hospital General, Segovia; Hospital Universitario Rio Hortega, Valladolid; Hospital Universitario Virgen de la Arrixaca, Murcia; Hospital Universitario Virgen de la Arrixaca, Murcia; Hospital Universitario Virgen de la Arrixaca, Murcia; Complejo Hospitalario San Millan-San Pedro, la Rioja

**Keywords:** hepatitis C, HIV, treatment, adolescents, pediatric, direct-acting antivirals

## Abstract

We evaluated the effectiveness and safety of direct-acting antivirals in adolescents with hepatitis C (HCV)/HIV coinfection using pooled individual patient-level data from 5 European cohorts. Of 122 participants in follow-up from November 2013 to August 2021, 19 were treated <18 years of age; of 15 with HCV RNA available at/after 12 weeks post-treatment, all had sustained virologic response with acceptable safety. This evidence addresses an important gap in knowledge of treatment outcomes in adolescents with HCV/HIV coinfection in real-life settings.

Children and adolescents living with HIV and hepatitis C (HCV) coinfection have higher risk of progressive liver disease than children with mono-HCV infection.^1–3^ Direct-acting antivirals (DAAs) for HCV treatment have cure rates of >90% among adults.^4^ The first DAA regimen approved for pediatric use by the European Medicines Agency was sofosbuvir/ledipasvir (SOF/LDV), approved for adolescents 12–17 years old in 2017. This was followed by pan-genotypic glecaprevir/pibrentasvir, approved for adolescents in 2019. In 2020–2021, approvals of some DAA treatments were extended to young children 3 years of age and above.^5^ However, there remains limited data on safety and effectiveness in children and adolescents, particularly those with HCV/HIV coinfection, despite being a priority group for DAA treatment.^2^ Recent case-series from Poland and Ukraine reported DAA use in 2 and 6 HCV/HIV coinfected adolescents respectively, all of whom achieved sustained virologic response (SVR).^6,7^ In this study, we describe the safety and effectiveness of DAAs in adolescents, using real-world data from a large observational pediatric HIV cohort collaboration in Europe.

## METHODS

The European Pregnancy and Paediatric Infections Cohort Collaboration includes 18 pediatric observational HIV cohorts across 15 countries in Europe and Thailand (https://penta-id.org/hiv/eppicc/). In brief, individual patient-level data are collected on children and adolescents in routine pediatric HIV care from HIV diagnosis or first entry to care through to the last visit and include demographic, clinical, laboratory and treatment data. Data were pooled using a modified HIV Cohorts Data Exchange Protocol. All cohorts have approvals from local ethics committees.

### Cohort and Participant Inclusion Criteria

Cohorts with data on ≥1 participant living with HCV/HIV coinfection who ever received DAA treatment after November 22, 2013 (date of first approval of DAAs in adults by the EMA^8^) were included in the study. This date was taken as a starting date to capture any early off-label use in the pediatric population. The latest date for the data cutoff varied across cohorts, from December 2020 to August 2021.

Participants within the selected cohorts who had positive HCV antibody at ≥18 months of age or HCV RNA at ≥6 months of age and were in follow-up during the defined period were included. Those <18 years of age and in follow-up after the DAA approval date were included in the analysis of DAA uptake. DAA treatment effectiveness was defined as the absence of quantifiable HCV RNA in serum after 12 weeks post-DAA treatment completion (SVR12). End of treatment (EOT) viral clearance was defined as undetectable HCV RNA at/after EOT and before week 12. Division of AIDS classification was used to grade the severity of alanine aminotransferase (ALT) and aspartate aminotransferase (AST) elevations, assuming an upper limit of normal of ≥30 U/L for ALT and ≥40 U/L for AST.^9–11^ Pretreatment values were the closest measurements available to DAA start date (from 6 months before, up to and including treatment start date). AST-to-platelet ratio index (APRI) and Fibrosis-4 score (FIB-4) at pre- and during treatment were calculated; an APRI score of ≥1.5 and a FIB-4 score of ≥3.25 were considered an indicator of significant and advanced fibrosis, respectively.^12^ Median [interquartile range (IQR)] of all markers are described, along with changes in division of AIDS grade or FIB-4 and APRI category during treatment. Post-treatment laboratory measurements were not available due to the short duration of follow-up post-treatment in data for analysis. Data analysis was conducted using Stata (17.0, StataCorp LLC, College Station, TX).

## RESULTS

Data from 5 cohorts in Spain, Poland and the Russian Federation were included in this analysis. The Spanish cohort is a multicenter cohort while the Polish cohort and the 3 cohorts in Russia are all single-center cohorts based in large pediatric infections referral centers.

**FIGURE 1.**
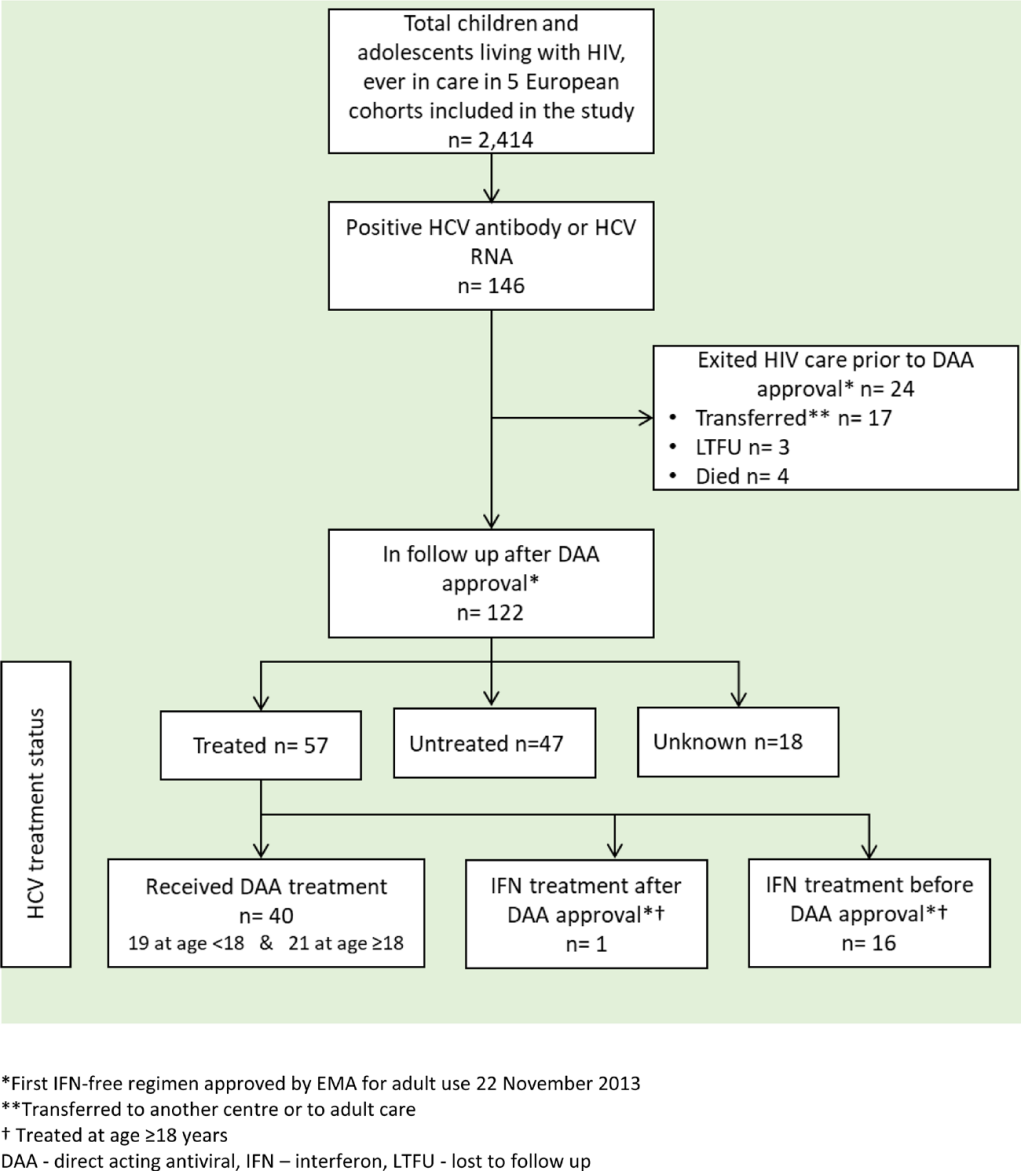
Participant selection from eligible cohorts in EPPICC. *First IFN-free regimen approved by EMA for adult use November 22, 2013. **Transferred to another center or to adult care. †Treated at age ≥18 years. DAA indicates direct-acting antiviral; IFN, interferon; LTFU, lost to follow-up.

Of 2414 children and adolescents ever in pediatric HIV care in the 5 cohorts, 146 (6%) had documented HCV diagnosis with positive HCV antibody or HCV RNA results at a median age of 6.3 [IQR 2.3, 9.0] years. Of these, 4 (3%) died and 20 (14%) exited the cohorts prior to the start follow-up date (Fig. 1). Of the 4 deaths, 1 was due to liver failure related to HCV at age 16.5 years; 2 deaths were unrelated to HCV and 1 had an unknown cause.

Of the 146 participants, 122 (84%) remained in follow-up after the start follow-up date and 90/122 (74%) were viremic. Nineteen participants had initiated DAA treatment <18 years old and 21 at age ≥18 years. One participant died without starting DAAs (not HCV related).

Among the 19 participants who started DAAs <18 years old, 12 (63%) were female, median [IQR] age of HCV diagnosis and DAA start was 6.3 [2.3, 9.0] and 15.8 [13.2, 16.3] years, respectively (Table 1). HIV RNA at DAA start (closest 6 months before to 1 week after) was available for 12 adolescents, 6 (50%) had viral load <50 copies/mL and 9 (75%) <1000 copies/mL. Sixteen participants (84%) received glecaprevir/pibrentasvir (all in Russia) and 3 (16%) SOF/LDV (2 in Poland and 1 in Spain). One participant started DAAs in 2016, 2 in 2019 and the remaining 16 in 2020. Duration of DAA treatment ranged from 8 to 16 weeks. All 19 adolescents completed their prescribed course of DAA treatment. One participant (5%) was reported to have an adverse event of a headache, considered related to SOF/LDV; the event resolved without treatment change.

**TABLE 1.** Characteristics of HCV/HIV Coinfected Adolescents Treated With Direct-acting Antivirals (n = 19)

	Demographic Characteristics/Pretreatment Laboratory Markers
	N (%) or Median [IQR]
Country of cohort	
Poland	2 (10.5%)
Russia	16 (84.2%)
Spain	1 (5.3%)
Sex, female	12 (63.2%)
Ethnicity, white European	19 (100.0%)
Mode of HIV acquisition	
Vertical	17 (89.5%)
Other	1 (5.3%)
Unknown	1 (5.3%)
Mode of HCV acquisition	
Vertical	5 (26.3%)
Unknown	14 (73.7%)
HCV genotype	
1b	8 (42.1%)
3	8 (42.1%)
4	3 (15.8%)
Age at HIV diagnosis	1.6 [1.2, 2.7]
Age at HCV diagnosis	6.3 [2.3, 9.0]
Age at DAA start	15.8 [13.2, 16.3]
ART regimen at DAA start	
2 NRTI + PI	7 (36.8%)
2 NRTI + NNRTI	7 (36.8%)
2 NRTI + INSTI	3 (15.8%)
Treatment interruption	2 (5.3%)
Unknown	1 (5.3%)
HIV RNA <50 copies/mL at DAA start (n = 12)	6 (50%)
CD4 cell count at DAA start (n = 18)	669 [560, 868]
ALT (n = 17)	
Nonelevated	10 (58.8%)
DAIDS grade 1 (mild elevation)	4 (23.5%)
DAIDS grade 2 (moderate elevation)	3 (17.7%)
Median [IQR] U/L	32 [27, 48]
AST (n = 17)	
Nonelevated	13 (76.5%)
DAIDS grade 1 (mild elevation)	4 (23.5%)
Median [IQR] U/L	29 [25, 48]
APRI (n = 15)	
<0.5 (no significant fibrosis)	12 (80.0%)
0.5–1.5	3 (20.0%)
>1.5 (significant fibrosis)	0
Median [IQR] score	0.32[0.24, 0.50]
FIB-4 (n = 15)	
<1.45 (no advanced fibrosis)	15 (100%)
Median [IQR] score	0.31 [0.24, 0.50]

AIDS indicates acquired immune deficiency syndrome; ALT, alanine transaminase; APRI, AST-to-platelet ratio index; ART, antiretroviral therapy; AST, aspartate aminotransferase; DAA, direct-acting antiviral; DAIDS, Division of AIDS; FIB-4, fibrosis-4; HCV, hepatitis C virus; INSTI, integrase strand transfer inhibitors; NNRTI, non-nucleoside reverse transcriptase inhibitors; NRTI, nucleoside/nucleotide reverse transcriptase inhibitors; PI, protease inhibitor; U/L, units/liter.

### Direct-acting Antiviral Effectiveness

SVR12 data were available for 15 of 19 (79%) adolescents; all achieved SVR12. Of the 4 participants with no SVR12 results available, 2 (11%) had EOT viral clearance and 2 (11%) had missing treatment outcome data.

### Changes in Laboratory Biomarkers and Fibrosis Assessments

Pre-DAA treatment ALT and AST measurements were available for 17 participants, of whom 7 (41%) and 4 (24%) had raised ALT or AST, respectively, all at grade ≤2 (Table 1). All normalized during treatment, except for 1 participant with no measurements available after treatment start. One participant with normal AST pretreatment had grade 1 raised AST during treatment. APRI and FIB-4 scores were available for 15 participants pretreatment (Table 1); none had scores indicating significant or advanced fibrosis either pre- or during treatment.

One participant was reported to have liver cirrhosis pretreatment, confirmed on liver biopsy, with transient elastography (TE, FibroScan) 14 kilopascals (kPa) at DAA start (age 16.8 years) and normal APRI and FIB-4 scores. This participant received SOF/LDV and achieved SVR12. At approximately 18 months after completion of DAA treatment, TE result increased to 25.9 kPa, although no clinical or laboratory liver disease decompensation was observed and HCV RNA remained undetectable.

## DISCUSSION

Children and adolescents with HCV/HIV are a priority group for treatment due to the higher risk of liver disease progression compared to their HCV mono-infected counterparts.^1–3^ This is one of the first studies reporting the effectiveness and safety of DAAs in adolescents with HCV coinfection. Despite the importance of treatment in this population living with coinfection, it is possible that a significant number of children and adolescents with HCV/HIV remained untreated for 7 or more years after DAAs were first approved in adults in Europe, reflecting the time-lag in regulatory approval and access to new treatments in the pediatric population. Among those treated, all were adolescents. All 15 participants with available HCV RNA results at/after 12 weeks post-treatment completion achieved SVR12. Due to 4 missing SVR12 results, the conservative estimation of effectiveness is at least 79% in this cohort. There were no reports of serious clinical or laboratory adverse events or adverse events leading to treatment change or discontinuation. Our conservative SVR12 results are lower than previously reported due to missing data in 4 participants, but among those with data, the DAA safety was consistent with case-studies reporting excellent safety in adolescents^6,7^ and adults with HCV/HIV coinfection.^13,14^ The favorable treatment outcomes support WHO and European recommendations to treat all children and adolescents with chronic HCV 3 years old and above, including those with normal liver function tests and no evidence of liver fibrosis.^5,15^ Further data on DAA treatment outcomes in younger children (preadolescence) are needed to confirm the safety and effectiveness.^5,15,16^

Overall, laboratory markers of liver function improved after start of treatment, with ALT and AST normalizing in all participants with raised pretreatment values. No participants were classified as having significant fibrosis using APRI and FIB-4 scores either before or during treatment, however, it is important to note that these measures have not been validated in children. Other limitations of the study include lack of data on DAA dosage and we did not assess change in HIV outcomes post-DAA treatment.

Importantly, 1 participant had cirrhosis at age 16 years, confirmed on liver biopsy. For this patient, elevated transaminases, ART and other factors could have caused an increase in the elastography results, nonetheless this highlights the importance of early treatment, ideally at younger ages to minimize the risk of HCV disease progression.

Despite the relatively small sample size, these data are, so far, the largest number of adolescents to date of DAA treatment in a pediatric HCV/HIV population, providing one of the first descriptions of real-world data on DAA use in this population. Further monitoring of the uptake of DAAs in the pediatric HCV/HIV population is needed to ensure children and adolescents are not left behind in the global HCV elimination campaign.^3^

## CONCLUSIONS

This study contributes to addressing an important gap in knowledge of treatment outcomes in adolescents with HCV/HIV coinfection in real-life settings. The favorable treatment outcomes observed support current WHO and European recommendations to accelerate DAA treatment for children and adolescents with HCV.

## ACKNOWLEDGMENTS

*We thank all the participants for their participation in these cohorts, and the staff members who cared for them.*

## Collaborating cohorts:

Poland: Polish pediatric cohort: Head of the team: Prof Magdalena Marczyńska, MD, PhD. Members of the team: Jolanta Popielska, MD, PhD; Maria Pokorska-Śpiewak, MD, PhD; Agnieszka Ołdakowska, MD, PhD; Konrad Zawadka, MD, PhD; Magdalena Pluta MD, PhD. Administration assistant: Małgorzata Doroba. Medical University of Warsaw, Poland, Department of Children’s Infectious Diseases; Hospital of Infectious Diseases in Warsaw, Poland.

Russia: The City HIV Centre, St Petersburg: Anna Samarina; St Petersburg Republican Hospital: Inga Latysheva, Liubov Okhonskaya, Vladimir Rozenberg, Evgeny Voronin; Irkutsk AIDS Centre: Alexksey A. Plynsky, Yulia Plotnikova.

Spain: CoRISPE-S and Madrid cohort: Receive funding from: Estudio del análisis clínico-epidemiológico de la infección por el vih en niños y adolescentes, mujeres embarazadas y sus hijos a nivel nacional. Ministerio Sanidad. Proyect 202007PN0002.

Paediatrics Units: María José Mellado, Luis Escosa, Milagros García Hortelano, Talía Sainz, Carlos Grasa, Paula Rodríguez (Hospital Universitario La Paz, Madrid); Pablo Rojo, Luis Prieto-Tato, Cristina Epalza, Alfredo Tagarro, Sara Domínguez, Álvaro Ballesteros (Hospital Universitario Doce de Octubre, Madrid); José Tomás Ramos, Marta Illán, Arantxa Berzosa, (Hospital Clínico San Carlos, Madrid); Sara Guillén, Beatriz Soto (Hospital Universitario de Getafe, Madrid); María Luisa Navarro, Jesús Saavedra, Mar Santos, David Aguilera, Begoña Santiago, Santiago Jimenez de Ory (Hospital Universitario Gregorio Marañón, Madrid); Amanda Bermejo (Hospital Universitario de Móstoles, Madrid); María Penín (Hospital Universitario Príncipe de Asturias de Alcalá de Henares, Madrid); Jorge Martínez (Hospital Infantil Universitario Niño Jesús, Madrid); Katie Badillo (Hospital Universitario de Torrejón, Madrid); Ana Belén Jiménez (Hospital Fundación Jiménez Díaz, Madrid); Adriana Navas (Hospital Universitario Infanta Leonor, Madrid); Eider Oñate (Hospital Universitario Donostia, Guipúzcoa); Itziar Pocheville (Hospital Universitario Cruces, Vizcaya); Elisa Garrote (Hospital Universitario Basurto, Vizcaya); Elena Colino, Olga Afonso (Hospital Insular Materno Infantil, Gran Canaria); Jorge Gómez Sirvent (Hospital Universitario Virgen de la Candelaria, Tenerife); Mónica Garzón, Vicente Román (Hospital General, Lanzarote); Raquel Angulo (Hospital de Poniente de El Ejido, Almería); Olaf Neth, Lola Falcón (Hospital Universitario Virgen del Rocío, Sevilla); Pedro Terol (Hospital Universitario Virgen de la Macarena, Sevilla); Juan Luis Santos, Álvaro Vázquez (Hospital Universitario Virgen de las Nieves, Granada); Begoña Carazo, Antonio Medina (Hospital Regional Universitario, Málaga); Francisco Lendínez, Mercedes Ibáñez (Complejo Hospitalario Torrecárdenas, Almería); Estrella Peromingo, María Isabel Sánchez (Hospital Universitario Puerta del Mar, Cádiz); Beatriz Ruiz (Hospital Universitario Reina Sofía de Córdoba); Ana Grande (Complejo Hospitalario Universitario Infanta Cristina, Badajoz); Francisco José Romero (Complejo Hospitalario, Cáceres); Carlos Pérez, Alejandra Méndez (Hospital de Cabueñes, Asturias); Laura Calle (Hospital Universitario Central de Asturias); Marta Pareja (Complejo Hospitalario Universitario, Albacete); Begoña Losada (Hospital Virgen de la Salud, Toledo); Mercedes Herranz,(Hospital Virgen del Camino, Navarra); Matilde Bustillo (Hospital Universitario Miguel Servet, Zaragoza); Pilar Collado (Hospital Clínico Universitario Lozano Blesa, Zaragoza); José Antonio Couceiro (Complejo Hospitalario Universitario, Pontevedra); Leticia Vila (Complejo Hospitalario Universitario, La Coruña); Consuelo Calviño (Hospital Universitario Lucus Augusti, Lugo); Ana Isabel Piqueras, Manuel Oltra (Hospital Universitario La Fe, Valencia); César Gavilán (Hospital Universitario de San Juan de Alicante, Alicante); Elena Montesinos (Hospital General Universitario, Valencia); Marta Dapena (Hospital General, Castellón); Beatriz Jiménez (Hospital Universitario Marqués de Valdecilla, Cantabria); Ana Gloria Andrés (Complejo Hospitalario, León); Víctor Marugán, Carlos Ochoa (Complejo Hospitalario, Zamora); Ana Isabel Menasalvas, Eloísa Cervantes, Beatriz Álvarez (Hospital Universitario Virgen de la Arrixaca, Murcia) and Paediatric HIV-BioBank integrated in the Spanish AIDS Research Network and collaborating Centers.

Adults Units: Cristina Díez, (Hospital Universitario Gregorio Marañón, Madrid). Ignacio Bernardino, María Luisa Montes, Eulalia Valencia, Ana Delgado (Hospital Universitario La Paz, Madrid); Rafael Rubio, Federico Pulido, Otilia Bisbal (Hospital Universitario Doce de Octubre, Madrid); Alfonso Monereo Alonso (Hospital Universitario de Getafe, Madrid); Juan Berenguer, Cristina Díez, Teresa Aldamiz, Francisco Tejerina, Juan Carlos Bernaldo de Quirós, Belén Padilla, Raquel Carrillo, Pedro Montilla, Elena Bermúdez, Maricela Valerio (Hospital Universitario Gregorio Marañón, Madrid); Jose Sanz (Hospital Universitario Príncipe de Asturias de Alcalá de Henares, Madrid); Alejandra Gimeno (Hospital Universitario de Torrejon, Madrid); Miguel Cervero, Rafael Torres (Hospital Universitario Severo Ochoa de Leganés, Madrid); Santiago Moreno, María Jesús Perez, Santos del Campo (Hospital Universitario Ramon y Cajal, Madrid); Pablo Ryan, Jesus Troya (Hospital Universitario Infanta Leonor, Madrid); Jesus Sanz (Hospital Universitario La Princesa, Madrid); Juan Losa, Rafael Gomez (Hospital Universitario Fundacion Alcorcon, Madrid); Miguel Górgolas (Hospital Fundacion Jimenez Diaz, Madrid); Alberto Díaz, Sara de la Fuente (Hospital Universitario Puerta de Hierro de Majadahonda, Madrid); Jose Antonio Iribarren, Maria Jose Aramburu, Lourdes Martinez (Hospital Universitario Donostia, Guipuzcoa); Ane Josune Goikoetxea (Hospital Universitario Cruces, Vizcaya); Sofia Ibarra, Mireia de la Peña (Hospital Universitario Basurto, Vizcaya); Víctor Asensi (Hospital Universitario Central de Asturias); Michele Hernandez (Hospital Universitario Insular, Gran Canaria); María Remedios Alemán, Ricardo Pelazas, María del Mar Alonso, Ana María López, Dácil García, Jehovana Rodriguez (Hospital Universitario de Canarias, Tenerife); Miguel Angel Cardenes (Hospital Universitario Doctor Negrin, Gran Canaria); Manuel A. Castaño, Francisco Orihuela, Inés Pérez, Mª Isabel Mayorga (Hospital Regional Universitario, Málaga); Luis Fernando Lopez-Cortes, Cristina Roca, Silvia Llaves (Hospital Universitario Virgen del Rocio, Sevilla); Maria Jose Rios, Jesus Rodriguez, Virginia Palomo (Hospital Universitario Virgen de la Macarena, Sevilla); Juan Pasquau, Coral Garcia (Hospital Universitario Virgen de las Nieves, Granada); Jose Hernandez, Clara Martinez (Hospital Universitario Clinico San Cecilio, Granada); Antonio Rivero, Angela Camacho (Hospital Universitario Reina Sofia, Cordoba); Dolores Merino, Miguel Raffo, Laura Corpa (Hospital Universitario Juan Ramon Jimenez, Huelva); Elisa Martinez, Fernando Mateos, Jose Javier Blanch (Complejo Hospitalario Universitario, Albacete); Miguel Torralba (Hospital Universitario, Guadalajara); Piedad Arazo, Gloria Samperiz (Hospital Universitario Miguel Servet, Zaragoza); Celia Miralles, Antonio Ocampo, Guille Pousada (Hospital Alvaro Cunqueiro, Pontevedra); Alvaro Mena (Complejo Hospitalario Universitario, La Coruna); Marta Montero, Miguel Salavert, (Hospital Universitario La Fe, Valencia); Maria Jose Galindo, Natalia Pretel (Hospital Clinico Universitario, Valencia); Joaquín Portilla, Irene Portilla (Hospital General Universitario, Alicante); Felix Gutierrez, Mar Masia, Cati Robledano, Araceli Adsuar (Hospital General Universitario de Elche, Alicante); Carmen Hinojosa, Begoña Monteagudo (Hospital Clinico, Valladolid); Pablo Bachiller (Hospital General, Segovia); Jesica Abadía (Hospital Universitario Rio Hortega, Valladolid); Carlos Galera, Helena Albendin, Marian Fernandez (Hospital Universitario Virgen de la Arrixaca, Murcia); Jose Ramon Blanco (Complejo Hospitalario San Millan-San Pedro, la Rioja).
